# Melancholia

**Published:** 1893-02

**Authors:** C. C. Howard

**Affiliations:** New York, N. Y.


### MELANCHOLIA.

C. C. Howard, M. D., New York, N. Y.

Case I.—Mrs. B——, æt. thirty-nine; dark hair, dark eyes, swarthy complexion; quick tempered, overbearing, proud; always enjoyed good health. Was suddenly seized with an irresistible desire, while sitting in the dentist's chair, to strike the dentist in the genital organs. This inclination became so strong that she excused herself and went home. It caused her much anxiety. She was afraid of some mental disturbance. She cried a good deal, became very restless. In about two weeks she went back to the dentist, having had no like impulse in the meantime. Was scarcely seated when the same irresistible impulse returned. It grew upon her so rapidly in the next ten days that every male she met, even her husband, her own sons, affected her the same way.

Her suffering became unbearable. About six o'clock every evening there would be a violent aggravation, lasting until bedtime, when she would generally fall asleep. During the aggravation she would walk the floor, wring her hands, and cry in the most pitiable manner. Or she would sit on the side of her bed, and sway herself backward and forward. Her countenance expressed the most abject suffering. She was sure she was not curable; she was becoming deranged, and wanted her children sent away so they would not see her.

She menstruated very profusely every three weeks; large, dark clots, with soreness of the vulva.

She did not apply to me for a month after the first manifestation.

Her haughty manner, swarthy complexion, the great mental anxiety, the wringing of the hands, and the swaying of the body, the sexual thoughts, together with the menstrual irregularities, led me to the selection of Platina, of which I gave her one dose of the 50mm.

She gradually improved for the next six weeks, menstruating at the twenty-eight-day period, everything being perfectly normal, when there was a sudden and violent return of the mental condition.

The relapse was accompanied by a quite marked disturbance of the stomach. As soon as the stomach was empty, she suffered from a gnawing hunger and griping pain, relieved by eating, but the eating caused an oppression.

While talking to her she received some flowers sent by a friend, and exclaimed, "How very kind, but take them out of the room, the odor is unbearable!" This symptom, with the condition of the stomach, relief from eating, and the mental restlessness, made me think of Graphites, of which I gave her one dose of the CM potency.

Now there was no change in her condition for twenty-one days, and, then, after a severe aggravation on the twenty-first night, she awakened completely relieved of all her symptoms.

In three months she became pregnant, and was well during the period of gestation, and gave birth to a healthy child.

About two months after labor a fear gradually came over her that she might do harm to her baby! This gave her so much anxiety that she would not be left alone with the infant. She was restless, melancholy, and tearful. There was a return of the stomach symptoms, and the same aggravation from the odor of flowers. She received another dose of Graphites, this time dissolved in twelve teaspoonfuls of water. She improved immediately, and has had no return up the present time—more than six months.

Case II.—Mrs. S——, æt. thirty-seven, mild, gentle disposition, easily worried, light complexion. Great tendency to grow fat; perspires on the slightest exertion and at night during her sleep. Last August had a very difficult labor, in which she sustained a severe laceration of the cervix, and a slight one of the perineum, which left her in a melancholy condition.

She was doubtful as to a future state; feared she was losing her reason, and that others would observe her confusion of mind. Loss of memory; dreaded seeing people—and left the room, if possible, when any one called. Very melancholy, depressed, and tearful. Continually worried over imaginary evils that might befall her family.

There was great difficulty in walking; the legs were stiff, gait tottering, knees would give out in walking, and she would fall unless support was near. There was dull, heavy, aching pain at the base of brain extending down the spine. Calc-carb.$^{cm}$, one dose. She began to improve in about ten days, and there was a complete restoration to health in two months.

### Tabes Dorsalis.

Case III.—Mr. A., æt. sixty-two, hard-working business-man of temperate habits, but never took any exercise. Had been sick for two years before consulting me, having had all sorts of allopathic abominations, even to the *hot iron*.

Presented the following conditions:—Inability to walk without looking at his feet—would stagger and fall in the dark, or on lifting his eyes from the ground. Walked with his feet far apart. Sensation as if walking on balls. Paroxysms of pain in thighs, legs, and feet; heavy, dull ache at times, and again sharp and lancinating. The paroxysms would last two or three days, and come about every week. They were worse in damp weather. There was a sensation in the feet as if being squeezed in a vise. This was particularly felt in the big toe of the left foot.

His appetite and digestion were good, although he had to be very cautious—eating to satiety would aggravate the pains. Craving for clean white rags. His bowels were very much constipated, and would only move when there was a large accumulation and with much straining. The feces were covered with mucus and streaked with blood. I gave one dose of Alumina$^{49m}$. Although I watched the case very carefully I could not see the slightest improvement for over nine weeks.

Then the bowels began to move more regularly and the stools assumed a more healthy appearance. Week by week the pains grew less and he walked with greater ease.

He gradually became able to walk in the dark, and after six months was practically well, and has remained so, up to the present time, about two years and a half after beginning treatment.

